# A hybrid effectiveness implementation trial testing an HIV and STI prevention program for mother figures and their adolescent girls and young women delivered by peer leaders: ZAIMARA study protocol

**DOI:** 10.1186/s12889-025-24332-5

**Published:** 2025-09-30

**Authors:** Geri R. Donenberg, Anjali Sharma, Tukiya Kanguya, Erin Emerson, Nok Chhun, Chowa Tembo Kasengele, Mable Mweemba, Mwamba Mwenge, Samuel Bosomprah, Gina Diagou Sissoko, Sybil Hosek, Carolyn Bolton Moore

**Affiliations:** 1 Center for Dissemination and Implementation Science, Department of Medicine, University of Illinois Chicago, Chicago, IL USA; 2 Centre for Infectious Disease Research in Zambia, Lusaka, Zambia; 3 Ministry of Health, Lusaka, Zambia; 4 Department of Biostatistics, School of Public Health, University of Ghana, Accra, Ghana; 5 Department of Medicine, University of Alabama at Birmingham, Birmingham, AL USA

**Keywords:** HIV prevention, Hybrid trial, Adolescent girls and young women, Zambia, Female caregiver-daughter intervention

## Abstract

**Background:**

Zambia is struggling to meet the 95-95-95 targets established by the Joint United Nations Programme on HIV/AIDS for adolescent girls and young women (AGYW), and HIV incidence remains high. The Zambian government has declared adolescent HIV prevention and sexual health as priorities, but new strategies are needed to facilitate HIV testing uptake among AGYW. Mother figures (MF), who are the center of Zambian families, may hold the key. The excessive burden on the healthcare workforce has impeded the implementation of effective HIV prevention programs. To sustain effective programs, implementation strategies are needed that reduce worker burnout and improve job satisfaction and employee retention. Task shifting to peer leader intervention delivery and attention to healthcare worker stress has the potential to strengthen and sustain program delivery. This study will test an intervention called ZAIMARA for AGYW and MF to improve HIV testing uptake and evaluate implementation outcomes when delivered by peer leaders.

**Methods:**

This 2-arm individually randomized hybrid effectiveness-implementation trial will compare ZAIMARA to a health promotion (HP) program across five sites in Lusaka. We will enroll up to 650 dyads comprised of 15-19-year-old Zambian AGYW and their MF. Peer leaders randomized and trained to deliver ZAIMARA or HP will receive mental health distress screening and referral versus nutrition and exercise screening. AGYW-MF dyads will participate in a 2-day group workshop and complete 6-, 12-, 18-, and 24-month assessments. Peer leaders will complete 6- and 12-month assessments. Primary outcomes are HIV-testing uptake for AGYW and job retention for peer leaders at 6-months. Secondary outcomes include incident sexually transmitted infections, including HIV, uptake of pre-exposure prophylaxis, and safer sexual behavior at 12-, 18-, and 24-months. We will evaluate implementation outcomes and intervention costs.

**Discussion:**

This study is poised to add a novel approach to HIV prevention among AGYW in Zambia and inform implementation considerations. ZAIMARA may provide a scalable solution to improve HIV testing and prevention practices for AGYW and identify features of implementation that strengthen sustainability. Findings will inform national HIV prevention strategies in Zambia and offer insights into the broader application of family-based interventions in similar low-resource settings globally.

**Trial registration:**

ClinicalTrials.gov, NCT06503666, Registered on 10 July 2024.

## Background

Zambia is unlikely to meet the 95-95-95 targets established by the Joint United Nations Programme on HIV/AIDS (UNAIDS) for adolescent girls and young women (AGYW) by 2030 [1]. HIV prevalence among 15–19 year-olds is 3.3%, with increasing incidence from 2010 to 2020 among AGYW [2]. Females are four times more likely to acquire HIV than their male peers (8.6% vs. 2.1%), AGYW HIV testing rates are low (45.4%), and 71.2% do not know their status. Of particular concern, over 63% of AGYW reported sexual activity, but only 37.1% used a condom at last sex [1–3]. Sexually transmitted infections (STIs) are a marker of risk for HIV, because they reflect unprotected sexual activity. The prevalence of STIs in Zambia is unknown because treatment is syndromic, and most STIs are asymptomatic. Thus, many STIs go untreated risking potentially serious reproductive health consequences, like infertility. Despite available public health services, including free HIV testing, prevention, care, and treatment, uptake remains suboptimal [4]. As a consequence, the Zambian government has declared adolescent HIV prevention and sexual health as key goals in its national strategy [5].

Unfortunately, barriers to HIV prevention, including cultural factors that discourage effective use of sexual and reproductive health services (SRH) in Zambia impede uptake [6]. AGYW may have fears and misconceptions of HIV and experience stigma surrounding adolescent sexuality. Anxiety can lead to a lack of social support from partners and peer groups that would otherwise be protective [7]. Families and communities are often the source of perceived stigma and reinforce inequitable gender and social norms that amplify girls’ vulnerability [6]. Similarly, health systems are frequently inaccessible and unfriendly to young people [8]. These intersecting barriers deter access to HIV and SRH services and long-term engagement in care.

As new HIV infections continue to outpace viral suppression, primary prevention remains the most viable strategy to stem the epidemic among Zambian AGYW. Yet, the effects of psychosocial interventions traditionally focused solely on adolescent behavior have been disappointing or relatively short-lived [9]. By contrast, programs that actively engage trusted family members in HIV prevention efforts may produce stronger and longer-lasting impacts, because they can address the sociocultural and structural drivers of HIV and challenge gender dynamics and HIV stigma in a safe and culturally appropriate venue [10–13]. In particular, mothers and other important female caregivers (aunts, cousins) (hereafter referred to as “mother figures” or MF) play a central role in AGYW’s sexual development and decision-making [14–18], and they can be role models for healthy relationships, open-communication, and self-care [19–24]. AGYW want to learn about sex from responsible adults, and mother figures can communicate prevention messages over time and tailor them to AGYW’s developmental stage. Family-based HIV prevention interventions can help AGYW and MF rethink gender roles and stereotypes [25], and MF may be a catalyst to promote AGYW safer sexual behavior, HIV testing, and PrEP uptake [25–27]. Hence, involving MF adds a new tool to the HIV prevention toolkit and can potentially improve the feasibility and acceptability of HIV testing and PrEP uptake.

Evidence for the effectiveness of family-based HIV prevention is growing. Programs that improve the quality of mother-daughter conversations about sexual decision-making, teach parents to use accurate and unambiguous information, and challenge cultural taboos that are barriers to mother-daughter communication about sex [10] can increase AGYW’s prevention behavior. Group-delivered family-based interventions can build on the power of collective and community support, change social and cultural norms to favor HIV prevention uptake, and create a sense of unity and collective efficacy to stem HIV infections among AGYW [28–30]. Yet, uptake and testing of family-based HIV prevention in Zambia has been slow, in part because involving adult family members in AGYW sexual health promotion challenges traditional values about adolescent pre-marital sexual behavior [31].

This study will adapt and evaluate *I*nformed *M*otivated *A*ware and *R*esponsible *A*dolescents and Adults (IMARA) for Zambian AGYW and their MF. IMARA was originally developed and tested in the United States with Black/African American 14–18-year-old AGYW and their primary female caregiver. A group randomized controlled trial in the U.S. showed reduced STI incidence among AGYW who received IMARA at 12-month follow-up compared to a matched control group [32], earning it the designation as a CDC “best evidence” HIV-prevention intervention. In 2019, we adapted IMARA for the South African context (IMARA SA), and findings from a pilot study revealed decreased AGYW mental distress [33], strong intervention feasibility, acceptability, and fidelity, and positive trends for increased South African AGYW’s HIV testing, PrEP uptake, and sexual risk reduction [34]. From 2022 to 2025, we systematically adapted IMARA for Zambia (i.e., *ZA*mbian *I*nformed *M*otivated *A*ware and *R*esponsible *A*dolescents and *A*dults; ZAIMARA) and carefully documented adaptations [35].

During the adaptation process, we recorded implementation determinants and processes informed by the Exploration, Preparation, Implementation, Sustainment (EPIS) Framework [36–39]. We identified a critical barrier to program sustainability, namely low staff retention. Like most resource-constrained settings, the Zambian healthcare workforce is overtaxed and understaffed. They report mental distress, emotional strain, and workplace burnout due to excess psychosocial demands [40]. Many healthcare workers leave their jobs prematurely due to low satisfaction, inadequate training, and feeling devalued, resulting in high costs for retraining and program continuity. To address this, we will use two implementation strategies, task shifting and mental health screening with referral, to reduce health worker burden and improve job retention. We will hire and train peer leaders to deliver ZAIMARA and evaluate the feasibility, acceptability, and fidelity of this alternative workforce. Research indicates that peer leaders can be trained to carry out a range of healthcare tasks [41–43] and deliver evidence-based interventions with fidelity with proper supervision [43]. We will also randomly assign peer leaders to receive monthly mental health screening and referral (where relevant) versus monthly nutrition and exercise screening and assess job retention at 6-months post-intervention.

## Overall study objectives

This study is a 2-arm hybrid effectiveness-implementation trial conducted in three phases. In Phase 1, which is complete, we systematically adapted IMARA for the Zambian context [35]. In Phase 2, we will evaluate ZAIMARA’s effectiveness on AGYW HIV testing, HIV and STI incidence, PrEP uptake, and sexual behavior. We will also assess the impact of monthly mental health screening and referral on peer leader job retention. In Phase 3, we will evaluate the acceptability, appropriateness, feasibility, treatment fidelity, sustainability, and costs associated with ZAIMARA.

## Methods/design

Aim 1 (IMARA adaptation completed. See Sissoko et al., under review).

### Aim 2 effect of ZAIMARA on AGYW HIV testing uptake at 6-months

**Overview**. AGYW and MF will be recruited from five geographically distinct communities and randomly assigned to the experimental (ZAIMARA) or control (health promotion) arm as a dyad. They will complete surveys at baseline, 6-, 12-, 18-, and 24-month follow-up using computerized assisted self-interview technology (CASI). AGYW will provide urine for STI screening, and we will offer HIV testing and pre-exposure prophylaxis (PrEP) at each time point. AGYW will receive healthcare free of charge at the site clinic as needed, including STI treatment in line with national guidelines. ZAIMARA and HP workshops will be delivered over 2-days (~ 12 h) and matched in time and intensity. Day 1 will last approximately 6-hours and include eligibility screening, consent/assent, the baseline survey, randomization, workshop day 1, and clinical assessments (HIV and STI testing, PrEP uptake). AGYW and MF will return to the site on a separate occasion (approx. 1 week later) for workshop day 2 of the intervention (~ 8 h). Sessions are conducted with AGYW and MF together and separately. To enhance the workshop experience and ensure participants’ comfort, light snacks, lunch and beverages will be provided during both workshops.

**Study sites**. The study will be conducted in five geographically distinct catchment areas in Lusaka, each feeding into a Ministry of Health (MoH) district clinic or hospital. The sites were chosen based on the high volume of adolescents seeking SRH services and the surrounding community population of AGYW. We expect enrollment of at least 2–3 AGYW-MF dyads per day per site.

**Recruitment.** Research staff, with assistance from neighborhood health committee (NHC) members will recruit AGYW and MF. NHC members are well-known in the community and recognized by the MOH as responsible for mobilizing and sensitizing communities about cross-cutting health issues, including research studies. The project coordinator will train NHC members to deliver recruitment messages using the recruitment brochures, and they will accompany outreach sessions. Recruiters will establish a landmark location in the neighborhood from which they can easily approach (or be approached by) women and AGYW. NHC members will initiate contact with a potential participant (AGYW or MF) and ask if she would like more information about the project. If yes, the research staff will explain the study and screen for inclusion/exclusion criteria. In addition, NHC members and research staff will implement a systematic door-to-door sensitization and recruitment strategy. Specifically, they will visit every fourth household within each selected neighborhood. During these visits, they will provide information about the study, address any questions or concerns from potential participants, and conduct preliminary screening to assess eligibility for study enrollment.

We will enroll approximately 16–20 dyads twice per month at each site for one year, beginning with two sites and then adding the remaining three sites. NHC members will receive approximately 50 kwacha (approx. 2 USD) for each eligible AGYW referred to the study to cover associated text/call costs and 50 kwacha to cover transport costs.

**Participants**. We will enroll up to 650 AGYW-MF dyads (120–130 dyads per site) for a total of 1200 to 1300 individuals. AGYW will be 15–19 years-old, female sex assigned at birth, unmarried, reside within the study catchment area, speak IciNyanja, ChiBemba, or English or a combination (the primary regional languages), willing and able to provide consent (≥ 18 years old) and/or assent (< 18 years old), and not known to have HIV (i.e., no positive test and not on ART). AGYW who participated in study Phase 1 or are enrolled in another sexual health intervention at the research site will be excluded. AGYW may or may not be sexually active; information about AGYW sexual activity will not be shared with MF. AGYW who test positive for HIV at baseline will complete the workshop, but then they will exit the study because it is not designed to provide support for persons with HIV. AGYW who become pregnant during the study may continue to participate.

MF are defined as an important and influential female in AGYW’s life (e.g., mother, aunt). MF will be chosen by the AGYW and agreed upon by the guardian/mother. MF must be ≥ 24-years-old, living with or in daily contact with the AGYW, speak IciNyanja, ChiBemba, or English or a combination, and willing and able to provide consent. MF who participated in study Phase 1 will be excluded.

**Consent/assent**. Study staff will explain research activities separately to MF and AGYW, allowing for independent consent and assent to prevent coercion. AGYW and MF must agree to participate as a dyad, but AGYW refusal will supersede MF consent. Consent/assent forms will explicitly state exceptions to confidentiality, for example, findings of suicidal ideation requiring referral, and these will be verbally reviewed with participants. The consent and assent documents will state that no information about AGYW sexual activity or HIV and STI test results will be shared with MF, reducing this barrier to consent/assent.

**Fig. 1:**
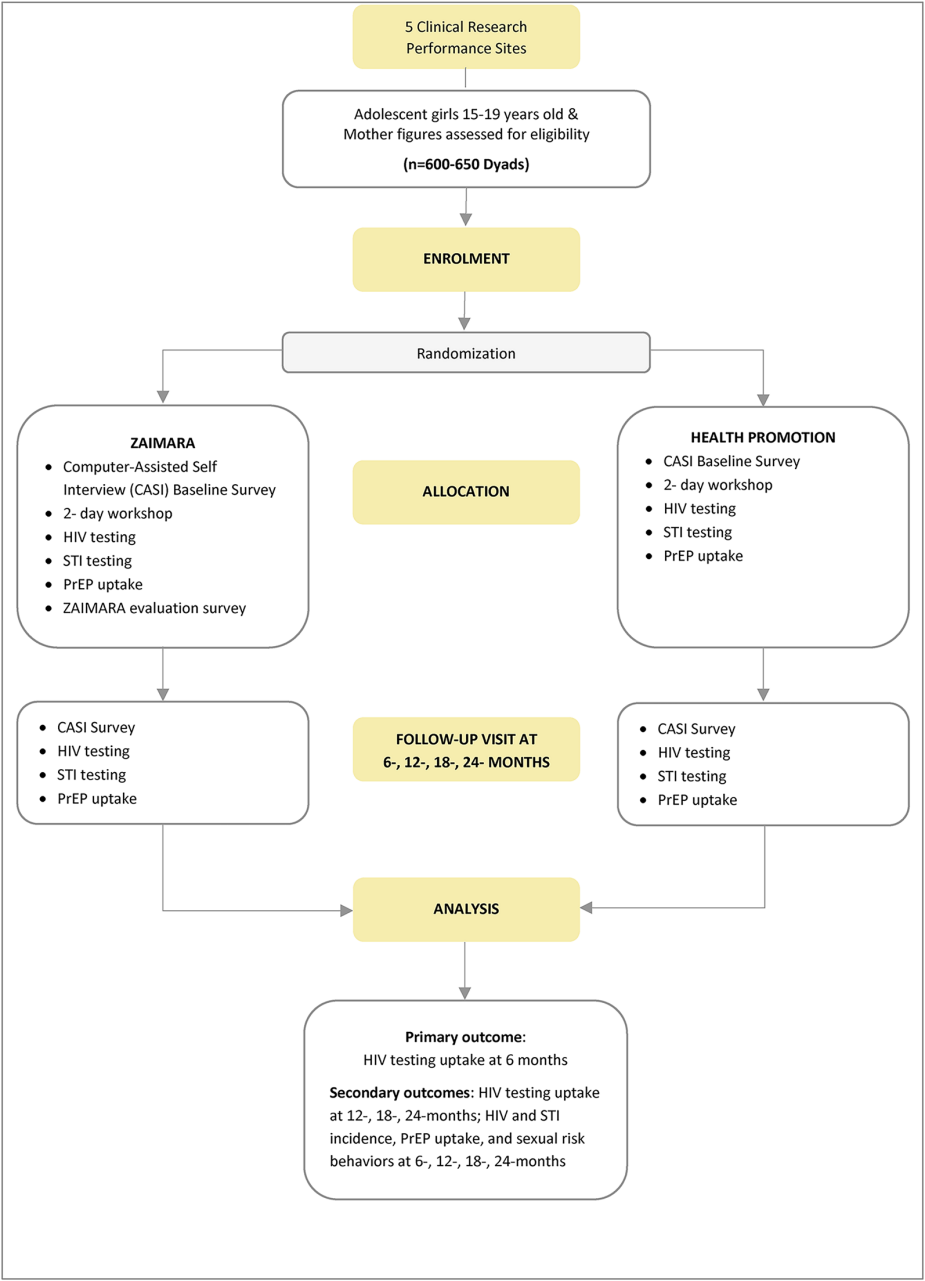
Flowchart for ZAIMARA intervention versus Health Promotion for AGYW-MF dyads

**Randomization**. We will randomize AGYW-MF dyads to one of two conditions (ZAIMARA or HP) following the baseline assessment (Fig. 1). We will use stratified permuted block randomization (blocks of 2) to ensure uniform distribution across arms and a balanced allocation (*n* = 300–325 ZAIMARA and *n* = 300–325 HP) throughout enrolment. AGYW and MF dyads will be randomized to the same condition. By stratifying the randomization process at the clinic level, we will account for potential variations in participant characteristics by neighborhood or clinic-specific factors, enhancing the validity and reliability of the study findings. Pre-determined randomization logs at each site will guide assignment to arms.

**Peer leaders**. Peer leaders (*N* = 60) will be female and live in the community where the program is delivered, have prior experience working with young people, have a minimum grade 12 education, and be proficient in reading and writing English and/or IciNyanja or ChiBemba. ZAIMARA and HP will be co-facilitated by four female peer leaders, each as follows: two peer leaders, ages 22–29 years-old will co-facilitate ZAIMARA to AGYW, and two peer leaders ages 30 years and older will co-facilitate ZAIMARA to MF. Likewise, two peer leaders ages 22–29 will co-facilitate HP to AGYW, and two peer leaders ages 30 years and older will co-facilitate HP to MF. Randomization will be stratified by clinic site, intervention arm, and age (22–29 years-old or ≥ 30 years-old).

**Training peer leaders.** Training for both interventions will stress the importance of following a standardized manual to ensure treatment fidelity across peer leaders. We will discuss group dynamics that may arise during the sessions and strategies to address them. Peer leaders will receive their curriculum in advance of the training to read and prepare questions for the in-person training. Training will occur over two weeks or approximately 30 h, led by two local ZAIMARA trainers who were trained by experienced IMARA SA facilitators during Phase I. During training week one, peer leaders will observe and act as group participants as the trainers demonstrate each activity in the curriculum. During week 2, the peer leaders will practice delivering the curriculum to each other. Two peer leaders will co-facilitate an activity while the remaining peer leaders observe and act as group participants. Peer leaders will rotate who delivers the intervention and who observes throughout the week so that all peer leaders have an opportunity to facilitate and observe. As peer leaders practice facilitating the interventions, the trainer and other peer leaders will provide feedback to improve delivery and strengthen treatment fidelity. The peer leaders will continue to practice until they are “certified” as competent (e.g., adherent, comfortable) to deliver ZAIMARA or HP by the trainers and investigators. We will conduct re-trainings as needed to prevent implementation drift. We will train new peer leaders using the strategies described above. They will observe one full intervention cycle by experienced peer leaders and participate in supervision prior to delivering the program.

**Supervision and quality assurance**. We will use a manualized curriculum and facilitator guide, weekly supervision, and observer ratings of adherence and competence to ensure treatment fidelity. Three locally trained nurses will provide weekly supervision to peer leaders separated by treatment arm. Two nurses will be trained to supervise ZAIMARA, and one nurse will be trained to supervise HP. Weekly supervision will include all trained peer leaders in the same treatment arm at the same site, even if they do not deliver the program (e.g., observers). Supervisors will review treatment fidelity forms in advance of supervisory sessions to identify potential challenges and areas lacking fidelity. They will refer to fidelity forms during supervision as needed. Supervisors and peer leaders will discuss session activities and problem-solve challenges. Weekly supervision will enhance quality assurance and quickly identify if a peer leader(s) deviates from the curriculum. In this case, the peer leader(s) will be re-trained until fidelity is achieved.

**Treatment fidelity.** All workshop sessions for both treatment arms will be observed by a trained peer leader who will be present during the intervention to assess treatment fidelity. Local ZAIMARA/HP trainers will observe 25% of the sessions. All observers will rate peer leaders on adherence and competence at the end of each workshop day. We will determine fidelity to ZAIMARA and HP by calculating the percentage of activities delivered (i.e., adherence) and peer leader mean competence rating.

## Intervention descriptions

**ZAmbian** **Informed** **Motivated** **Aware and** **Responsible** **Adolescents and Adults (ZAIMARA)**. IMARA (see Table 1) was derived from three CDC-designated evidence-based interventions: SISTA (Sisters Informing Sisters about Topics on AIDS) [44], SiHLE (Sistering, Informing, Healing, Living, and Empowering) [45], and Project STYLE (Strengthening the Youth Life Experience) [46]. IMARA engages AGYW and their MF to address the individual, sociocultural, and structural factors that influence AGYW’s sexual behavior [32]. Ideally ZAIMARA is delivered in groups of 6 to 10 participants, with certain activities conducted separately for MF and AGYW, while others are implemented jointly. The separate MF and AGYW groups run simultaneously, covering parallel content. Joint activities are designed to enhance MF’s credibility as a resource for HIV/STI prevention and facilitate the practice of new skills. Additional activities focus on conflict negotiation, assertive communication, and strengthening the AGYW-MF relationship. The material is presented in a group format to promote structural change, build community norms for prevention, and reduce HIV stigma.

Beginning with an icebreaker and/or a national poem to enhance Zambian and gender pride, facilitators then review ZAIMARA’s goals. The program motto is presented to emphasize strong AGYW-MF relationships, foster sisterhood and community empowerment, build group cohesion, and increase motivation for HIV/STI prevention [47]. At the start of the workshop on day 1, each MF and AGYW signs the ZAIMARA pact to confirm their commitment to the program. ZAIMARA spans approximately 10–12 h, delivered over two days, ideally separated by one week to allow participants time to assimilate information. At the end of workshop day 1, MF and AGYW are given homework to complete before workshop day 2. ZAIMARA weaves in the impact of mental health issues and alcohol and drug use on HIV/STI risk (e.g., condom use while inebriated) throughout the curriculum.

**Table 1:** Overview of ZAIMARA

Theory	Selected IMARA activities
***Individual factors***	
Knowledge about HIV, STI, PrEP, HIV testing and attitudes about PrEP	Facts of HIV/AIDS/STI/PrEP/HIV Testing; Prevalence and incidence for women; Perceived vulnerability; Personal risk triggers (people, places, feelings) and risk plans; Demonstrate and practice condom use; Importance of regular HIV testing and PrEP use where appropriate to risk behavior
Mental health	Destigmatize mental health; Links between mental health problems and HIV risk behavior; Recognize how feelings trigger risk-taking; Teach healthy coping strategies; Referrals for counseling
***Social factors***	
Family context	Importance of strong AGYW-MF relationships, and open and positive communication; Review need for MF monitoring; Create personalized monitoring plans; Understand the impact of mental health on HIV/STI-risk; Increase AGYW-MF warmth and positive interactions; Teach and practice effective communication with partners and family
Geographically constrained networks	Discuss HIV/STI risk in the context of neighborhood and sexual networks
***Structural factors***	
Gender dynamics	Expectations of women; Media images; Healthy and unhealthy relationships; Gender Based Violence; Gender role expectations and stereotypes; impact of gender of dynamics on HIV/STI risk
Partner relationships	Implications of age discrepant partners and concurrent partnerships; Importance of HIV testing, STI testing and treatment, and PrEP use and persistence; Communication with partners about HIV testing and PrEP
HIV stigma and discrimination	Changes in community norms to reduce stigma toward HIV/STIs, PrEP, and HIV testing

**Health promotion (HP): Time- and attention-matched control**. HP will be delivered as the comparison program and was used as the control group in IMARA SA. HP focuses on promoting healthy living by encouraging good nutrition, regular exercise, and informed consumer behavior. Although the HP curriculum does not explicitly address HIV/STI prevention, it includes basic information about HIV and other STIs due to the high-risk for AGYW. Like ZAIMARA, HP uses interactive games, videos, worksheets, and group discussion and MF and AGYW will meet in separate groups and together.

**Contamination**. Contamination across arms will be minimized as follows. (1) Peer leaders will not overlap, and training will occur separately. (2) ZAIMARA and HP will occupy different spaces at the site to reduce participant contact.

**Retention.** At baseline, we will obtain AGYW and MF phone numbers, home address (and draw a map), and other contact information (phone/text/WhatsApp). We will collect the phone numbers, home addresses, and text/WhatsApp information for at least two persons who will always know how to reach them in the future. We will make reminder calls the day before each workshop day and follow-up assessments to confirm attendance, and we will update our locator information at each contact. If the phone number/text/WhatsApp is disconnected, we will contact people from participants’ records. If AGYW were recruited through a clinic site (vs. outreach), we will seek assistance from the health facility and NHC members to locate them. Research assistants will visit the AGYW’s and MF’s last known address to try to locate them in-person. If the AGYW and/or MF no longer live at the address, we will ask their contacts to help us locate them. We will continue to try to locate them until the window for their assessment closes.

**Study assessments.** AGYW and MF will complete 1.5-hour surveys via CASI at baseline, 6-, 12-, 18-, and 24-months post-intervention, and research staff will be present to answer questions and assist participants as needed. AGYW will provide a urine sample to test for three STIs (gonorrhea, chlamydia, and trichomoniasis). At the end of workshop day 1, AGYW will be offered HIV testing and PrEP (where appropriate). AGYW who want and are eligible for PrEP will receive a one-month supply or referral for PrEP depending on their preference and available supplies. They will also be referred to PrEP services closest to them for refills. AGYW may refuse HIV and STI testing and/or PrEP and remain in the study. AGYW and MF will each receive K200 (~$9.00) on both workshop days and each follow-up assessment, K200 for transport, and other incentives (e.g., T- shirts, sanitary towels, hygiene products such as soap at each visit) during in-person visits.

### Demographics

AGYW and MF will report their age, education, employment, income, food and housing security, marital status, and number of children. AGYW who report being married at baseline will be excluded.

### Primary outcome

AGYW uptake of HIV testing at 6-months post-intervention.

### Secondary outcomes

AGYW uptake of HIV testing at 12-, 18-, and 24-months. At 6-, 12-, 18-, and 24-months, AGYW PrEP uptake; HIV and STI incidence (gonorrhea, chlamydia, and trichomoniasis); Self-reported sexual behavior (e.g., condom use, substance use during sex, number of partners, concurrent partners) [48]. Standard cost evaluation methods [49, 50], including personnel time and non-personnel resources (e.g., travel expenses, snacks, incentives, curricular materials).

**Mediators and moderators**: At baseline, 6-, 12-, 18-, and 24-month follow-up: AGYW and MF reported HIV and STI knowledge [51], and PrEP knowledge and attitudes [52], parental acceptance and rejection in the AGYW-MF relationship [53], and quality and quantity of AGYW-MF communication [54–56]. AGYW will indicate their mental health and trauma exposure [57–60], substance use [61–63], and partner dynamics and communication [56, 64, 65].

**Sample size.** The sample size (*N* = 600–650 AGYW-MF dyads; *N* = 1,200–1,300 participants) was determined based on the primary hypothesis that HIV testing at 6-months will be higher among AGYW who receive ZAIMARA versus HP. Assuming 10% attrition at 6-months, we will have a sample size of *n* = 270 AGYW in ZAIMARA and *n* = 270 AGYW in HP. With at least 80% power, a 2-tailed significance level of 0.05, and the assumption of proportions in ZAIMARA and HP of 0.454 (null hypothesis), we will be able to detect a difference of 0.12 between the group proportions (equivalent to risk ratio = 1.26).

Effect sizes for binary outcomes is given in terms of the relative risk (RR), where RR = 1 indicates no treatment effect. A RR < 1 indicates a protective effect of the intervention. With 80% retention, we will have ≥ 80% power to detect RRs ≤ 0.38 when the proportion in the control group is 20%, and RRs ≤ 0.65 when the proportion in the control group is 50%. We calculated the standard effect size (*d*) in standard deviations for group mean comparisons with 80% power for continuous outcomes, where *d* = 0 is defined as no treatment effect. We will be able to detect a small to medium effect size (*d* = 0.36) even with 75% retention. Low HIV incidence among AGYW (2.54%) per year will allow us to explore incidence, but we do not expect sufficient statistical power to test intervention effects on HIV incidence.

### Aim 3 impact of monthly mental health screening with referral on peer leader job retention

**Overview.** Peer leaders will be individually randomized within site and arm (ZAIMARA vs. HP) to receive a monthly mental health screening plus referral for services where relevant versus monthly nutrition and exercise screening with instructions for improvement where relevant. We will compare job retention at 6-months (primary outcome) and job satisfaction at 6- and 12-months.

**Participants**. All peer leaders (approximately *N* = 60 plus any new hires during the study) will be eligible to participate.

**Recruitment.** We will invite all peer leaders to participate in the study prior to training. The study coordinator will contact each peer leader, explain the study, and request participation. All peer leaders will be employed and paid by the study for non-research activities.

**Consent**. Peer leaders who express interest in participating in the study will engage in formal consent procedures by research assistants prior to the baseline assessment. Peer leaders who do not want to participate in the research will be trained to deliver the intervention and participate in supervision but will not contribute study data.

**Retention.** At baseline, we will obtain peer leaders’ phone numbers, home addresses (and draw a map), and other contact information (phone/text/WhatsApp). We will also collect the phone numbers, home addresses, and text/WhatsApp information for at least one other person who will always know how to reach them in the future. At least one month before each follow-up assessment, we will contact peer leaders to confirm the appointment. We will attempt to retain all peer leaders even if they resign from their employment with the study. If we do not reach the peer leader after one week and at least three attempts, we will follow several steps: 1) If the phone number/text/WhatsApp is disconnected, we will contact people from peer leaders’ records; (2) If peer leaders were recruited through any other projects, we will seek their assistance to locate them; (3) Research assistants will visit peer leaders’ last known address to try to locate them in-person. (4) If peer leaders no longer live at the address, we will ask their contacts to help us locate them.

**Fig. 2:**
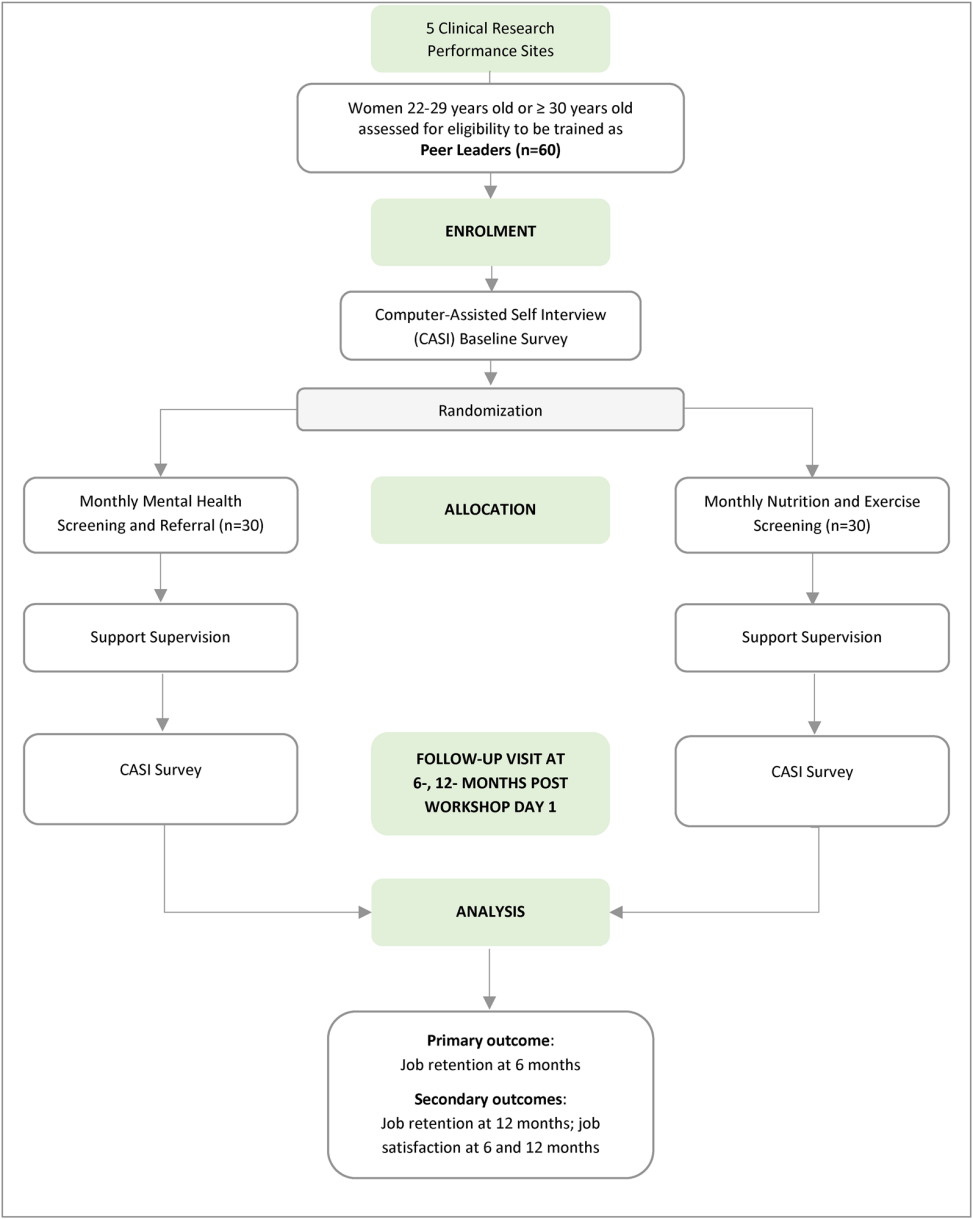
Flowchart for Peer Leader Mental Health Distress Screening versus Nutrition and Exercise Screening

**Randomization.** We will randomize peer leaders to one of two conditions, mental health distress screening with referral (*n* = 30) or nutrition and exercise screening with improvement suggestions (*n* = 30) prior to the first workshop at the site but after the baseline assessment (Fig. 2). We will stratify peer leader randomization by treatment arm (ZAIMARA and HP) and age (22–29 years-old or ≥ 30 years-old) using predetermined block size.

**Monthly health screening procedures.** During the 4th supervision session each month, peer leaders will complete a standard screening of their mental health distress or nutrition and exercise on a handheld computer tablet. The weekly supervision session and the screening will be conducted separately. Peer leaders whose mental health screen indicates distress will receive a prompt with a referral for additional evaluation by a mental health professional. Peer leaders who report sub-optimal nutrition and exercise will receive a notification immediately following their screening with instructions for improvement (see below). The study nurse will collect the tablets and document peer leader scores and referrals.

**Screening measures**. Peer leaders randomized to the mental health distress screening and referral condition will complete the *Kessler Psychological Distress Scale*−5 (*K5*), a 5-item self-report measure of psychological distress over the past four weeks [66]. Peer leaders who screen positive for mental health distress (≥ 11) will receive a prompt with a referral for additional evaluation and support by a mental health professional. Peer leaders randomized to nutrition and exercise will complete two sections about nutrition and physical activity on the *Fantastic Lifestyle Checklist* [67]. Peer leaders who report sub-optimal nutrition and exercise (≤ 13 on *Fantastic Lifestyle Checklist*) will receive a prompt to examine areas where they scored 0 and 1 and choose one area to change. Once they make their selection, they will receive tips on how to change, including seeking advice from the health care clinic or a personal trainer.

**Exit interviews.** If a peer leader resigns, we will conduct an exit interview to understand their reasons for leaving. Interviews will be conducted by a trained research assistant addressing themes associated with job satisfaction, self-efficacy, support, and workplace stress. This will inform future peer leader-led interventions.

**Study assessments**. Peer leaders will complete a ~ 40-minute baseline survey via CASI pre-training, pre-workshop day, and 6-, and 12-months after the first workshop. They will receive K200 (~$9.00) at the end of each assessment plus K200 (~$9.00) transport funds. Assessments will be administered by research assistants blinded to study arm. For peer leaders who are no longer employed by the study, we will be flexible in the location (remote or in-person) of data collection. All peer leader data will be de-identified prior to review by investigators and the study coordinator to preserve confidentiality and eliminate fear of recrimination, except if they endorse severe distress, in which case they will be evaluated and referred (as needed) for care.

#### Demographics

Peer leaders will report their age, marital status, number of children, education, food security, and stable housing.

#### Primary outcome

The percentage of peer leaders retained at 6-months.

#### Secondary outcomes

The percentage of peer leaders retained at 12-months; Job satisfaction [68] at 6- and 12-month follow-up.

**Mediators and moderators**: At baseline, pre-workshop day 1, and 6- and 12-month follow-up, peer leaders will report their confidence delivering sex education (*Sex Education Confidence Scale* (SECS)) [69], HIV and STI knowledge [51], job satisfaction (*Satisfaction of Employees in Health Care (SEHC))* [70], PrEP knowledge and attitudes [52], depression (Patient Health Questionnaire-9 [58]), anxiety (GAD–7 [71]), trauma (PC-PTSD-5 [72]), alcohol use (AUDIT C [63], and drug use (ASSIST 3 [62]). Peer leaders who screen positive for clinically significant distress (GAD-7: ≥ 10; PHQ-9: ≥ 10; PC-PTSD5 ≥ 3) or alcohol and substance use disorder (AUDIT C: ≥ 3; ASSIST3 ≥ 4) at any assessment will be referred for further evaluation and treatment at the closest government facility. If peer leaders report suicidal ideation, they will be immediately referred to counseling.

### Aim 4 ZAIMARA implementation outcomes

**Overview**. We will use a combination of surveys, source documentation, focus groups, and individual interviews to evaluate the acceptability, appropriateness, feasibility, fidelity, sustainability, and costs associated with ZAIMARA.

**Participants**. Participants will comprise six different groups representing each clinic site in the study: AGYW (*n* = 15), MF (*n* = 15), peer leaders (*n* = 10), research staff (*n* = 10) (i.e., local investigators, study coordinator, research nurses, research assistants, data manager, M&E coordinator), clinic personnel (*n* = 10) (i.e., nurses providing HIV and/or STI or other health services to adolescents, department heads), and other key partners (*n* = 15) (i.e., Ministry of Health, Technical Working Group, community leaders, religious leaders, local councilors, and Community Based Organization representatives). To be eligible, AGYW and MF must have participated in the effectiveness trial and the 24-month follow-up. For all others, a willingness to consent is required. All participants must have knowledge of ZAIMARA, its goals and objectives, and its procedures.

**Recruitment.** We will invite research staff and clinic personnel at all five sites to complete implementation surveys before workshop day 1 and again at 6-, 12-, and 24-month follow-ups. We will invite peer leaders who deliver ZAIMARA, research staff and clinic personnel, and key partners to participate in separate focus group discussions (FGDs) at the completion of the study. We will invite select partners, AGYW, and MF to participate in individual in-depth interviews (IDIs) remotely or in person. Participants will complete informed consent prior to data collection. The consent process will emphasize that study engagement is voluntary and will have no impact on their employment or the ability to receive services at the site. Exceptions to confidentiality as required by law will be explained.

**Procedures.** We will use a combination of self-report, monitoring logs, attendance records, clinical information, and structured observations to assess ZAIMARA’s implementation. FGDs (60–90 min) and IDIs (45–60 min) will be conducted at the end of the study by an experienced qualitative researcher in a quiet, private space in the participant’s preferred language (national or local) and using a semi-structured guide. IDIs and FGDs will be audio-recorded, and field notes will be taken during the interviews to characterize non-verbal cues, body language, and context. Using Proctor’s Implementation Outcomes Framework [73], we will conduct a qualitative investigation of ZAIMARA’s acceptability, appropriateness, fidelity, feasibility, sustainability, and cost effectiveness to improve future integration and intervention uptake [73–75]. The qualitative research assistants will maintain audio-recordings, notes, and analytical memos. We will provide a transport refund of K200 (~$9.00) to all participants. Table 2 indicates the implementation measures, theoretical framework, data source, and the time points for data collection.

**Table 2:** Implementation research measures, timepoints, source material

Topic	Items	Timepoint(s)	Source material
Reach	3	Completion of enrolment	Neighborhood Health Committee Members, Research Staff, Peer Leaders
Reach	1	Completion of enrolment	% of eligible AGYW enrolled in ZAIMARA. Catchment area estimates and enrolment numbers
Adoption	13	Pre-workshop day 1; 6-mo; 12-mo	Peer leaders
Adoption	13	Pre-workshop day 1; 6-mo; 12-mo; 24-mo	Clinic personnel; Research staff
Adoption	1	Study completion	% of sites and MoH staff intending to adopt ZAIMARA
Implementation	3	Pre-workshop day in the site 1; 6-mo	Peer leaders
Maintenance	16	Pre-workshop day in the site 1; 6-mo; 12-mo; 24-mo	Clinic personnel; Research staff; Community sensitization
Maintenance	16	Pre-workshop day 1; 6-mo; 12-mo	Peer leaders
Feasibility	6	6-mo	Peer leaders
Feasibility	9	Workshop day 2	AGYW, MF
Acceptability	5	6-mo	Peer leaders
Acceptability	6	Workshop day 1 and 2	AGYW, MF
Appropriateness	6	Workshop day 2	AGYW, MF
Appropriateness	6	6-mo	Peer leaders; Research staff; Clinic personnel
Fidelity	17	Workshop day 1 and 2; 25% reliability check	Peer leader observers; Local trainers
Sustainability	9	12-mo, 24-mo	Research staff
Cost effectiveness	NA	Study completion	Study records, expenditures, spread sheet

### Data analyses

**Overview.** We will use a mixed-methods convergent parallel design to explore the effectiveness of ZAIMARA in increasing HIV testing in 15-19-year-old AGYW, and the implementation reach, adoption, appropriateness, acceptability, feasibility, fidelity, maintenance, evaluation, and cost-effectiveness. We will analyze qualitative and quantitative data separately, place findings side by side on a results matrix to triangulate findings and examine how they converge/diverge and relate to each other to determine the completeness and strength of the evidence.

**Missing data**. We expect missing data to result from subject attrition. We expect at least 80% retention at 6-months (the primary outcome point) based on prior research. We will examine if AGYW who complete the follow-up assessments differ systematically on baseline data from those lost to follow-up. In the absence of evidence to the contrary, missing values will be regarded as missing at random (a function of observed outcomes and covariates), and we will use all available data (including non-completers) in analyses. We will conduct a sensitivity analysis to quantify potential bias due to non-ignorable dropout.

## Data management

All participants will be assigned a unique identification number. Survey data will be collected using REDCap (Research Electronic Data Capture), a web-based platform [76, 77], and stored in password-protected cloud-based system and secure server maintained at the Centre for Infectious Diseases Research in Zambia (CIDRZ). A research nurse at each site will collect laboratory data and record results in a REDCap file separate from personal identifiers and survey data. Data will be accessible only to study personnel. Bolton Moore (MPI) and Hosek (MPI) will review requests to access the data, and data files will be de-identified prior to sharing with others. Study investigators will check for data completeness and accuracy, and the confidentiality of records will be upheld.

### Statistical analysis

Preliminary analyses will summarize baseline characteristics of AGYW, MF, and peer leaders, generate frequencies (proportion) for categorical variables, medians (interquartile range) for continuous variables, and summary statistics of predictor, outcome, and mediating/moderating variables to screen and clean the data. We will evaluate randomization integrity by examining the distribution of baseline characteristics between randomization groups (i.e., AGYW assigned to ZAIMARA versus HP, peer leaders assigned to ZAIMARA versus HP, and peer leaders assigned to screening (mental health distress versus nutrition/exercise). We will adjust for potential confounders not balanced at baseline. We will examine if AGYW and peer leaders who complete the follow-up assessments differ systematically at baseline from those lost to follow-up. We will evaluate correlations among the variables, inspect scale reliabilities, and create summary scores. We will use models with random intercepts to account for clustering (if needed) and test the significance of clustering of subjects within groups and within sites.

**Aim 2**. The primary analysis will be based on the intention-to-treat population. We will use a two-sided z-test with unpooled variance to test the inequality of the proportion of HIV testing uptake at 6-months among AGYW who receive ZAIMARA compared to AGYW who receive HP. We will use log-binomial generalized linear model to estimate the effect of ZAIMARA on proportion with HIV testing uptake at 6-months, adjusting for potential confounders. Likelihood p-value < 0.05 will be considered statistically significant. We will evaluate the intervention effect on secondary *binary* outcomes (e.g., PrEP uptake, incident STI) in an analogous way. We will perform subgroup analyses, using test of interaction [78, 79] to evaluate if the effect of ZAIMARA on the primary outcome (HIV testing) differs systematically depending on hypothesized variable, such as mental health (e.g., depression, anxiety). We will include interactions between treatment condition and subgroup covariates in the generalized linear model, and obtain effect estimates, 95% confidence intervals, and p-values. For *continuous* secondary outcomes (e.g., HIV/STI knowledge score), we will evaluate the effect of ZAIMARA using linear regression models.

**Aim 3.** We will summarize baseline characteristics of peer leaders by group using frequency and proportion for categorical variables while median and interquartile interval will be used for continuous variables. The primary analysis will be based on a comparison of the proportion of peer leaders retained in their positions, defined as present at 6 months; never absent without leave, between the mental health distress screening and referral versus nutrition and exercise screening groups using two-sided z-test with unpooled variance. All analyses will be performed using Stata 18 MP8 (StataCorp, College Station, TX, USA). We will assess mental health at baseline and end of term for both groups.

**Aim 4.** We will estimate the implementation outcomes for the ZAIMARA intervention. We will summarize all scales using mean score and standard deviation. We will calculate the mean response across items for each implementation outcome, separately for each group of respondents. We will calculate overall adoption rate as number (%) of sites and MoH staff intending to adopt ZAIMARA. We will evaluate fidelity to ZAIMARA by calculating the percentage in which the peer leaders adhered to the curriculum (e.g., 80%) and how competent they were based on mean ratings.

Qualitative data will be evaluated using thematic analysis with a phenomenological approach. We will record, transcribe, and enter transcripts into NVivo (NVivo qualitative data analysis software; QSR International Pty Ltd. Version 12, 2018). We will format analytical memos and oversee development of the codebook based on determinants in the Implementation Research Logic Model (IRLM) and EPIS phases. Research assistants will complete analytical memos after each IDI/FGD to iteratively inform the next round of data collection. Through periodic assessment for thematic saturation, two study qualitative analysts will finalize the codebook containing deductive codes arising from the IRLM (e.g., inner setting) and inductive codes that emerge from the data (e.g., cultural taboos, rules of communication), resolving differences in team meetings. We will check interpretation within 7 days of data capture with the data collectors to ensure the correct meaning is captured. Next, the findings will be presented to the study nurse and peer leaders followed by presentation to ZAIMARA recipients for member checking.

**Cost data analysis**. The cost analyses will examine the costs of ZAIMARA with respect to HIV/STI acquisition, the net cost of the intervention to the health system adjusting for cost savings from STI reduction. We will explore HIV incidence where possible. We will conduct individual cost effectiveness (CE) analyses for all 3 STIs and assess the overall CE of ZAIMARA. In addition, we will conduct 2 supplementary analyses to estimate: (a) the CE of ZAIMARA when viewed strictly as an HIV prevention intervention; and (b) the CE with all 4 STIs (G, C, T, HIV) included.

### Data monitoring

The CIDRZ internal quality control mechanisms will be used to monitor data. Monitoring will include weekly reviews of participant records, consent and assent documents, source documentation, data collection instruments, screening/enrollment logs, visit checklists, baseline checklists, chart notes, referral forms, participant evaluation forms, study blood draws, and survey database entry. We will assemble a Data Safety and Monitoring Board to review our activities, ensure participant safety, and evaluate findings in an interim analysis to determine if the randomized controlled trial should continue or be stopped.

### Adverse event monitoring

We will monitor, document, and report adverse events and study harms. Events will be reported within 24 h of becoming aware to the study’s Multiple Principal Investigators and to the Institutional Review Boards according to national and local guidelines.

### Dissemination plans

We will disseminate study results using tailored approaches designed for different audiences, including printed documents, online reporting, peer-reviewed publications, conference presentations, the CIDRZ website and social media platforms (Facebook, Twitter, Linkedin), quarterly reports to the District Health Office and MOH, Zambia National Public Health Institute, and civil society organizations. We will share findings with AGYW and MF participants, CIDRZ adult and adolescent advisory boards, community members through community sensitizations, and at a monthly meeting of the National Health Research Authority. We will conduct a dissemination workshop within the first 6-months of closing the study to share what was planned, implemented, achieved, and steps forward.

## Discussion

The Zambian government has declared adolescent HIV-prevention and sexual health as priorities in its national strategy and is seeking innovative approaches to improve awareness and utilization of available services [5]. Multilevel comprehensive prevention approaches that are integrated and tailored to the local epidemiology and cultural context are more likely to achieve and sustain maximum reductions in STI/HIV-risk among AGYW. This is especially true for AGYW whose persistent HIV disparities are explained by a multiplicity of social and structural inequities that shape and constrain HIV-risk behavior and continue to drive incident infections. ZAIMARA will add a new strategy to the prevention toolbox by leveraging the AGYW-MF relationship and communication to promote positive SRH, including uptake of HIV testing and PrEP in Zambian AGYW.

Family-based prevention is particularly important as incident HIV increases precipitously among AGYW 19–24 years [80] signaling the need to intervene at an earlier age. ZAIMARA is based on successful models implemented in the U.S. (i.e., IMARA [32]) and in South Africa (i.e., IMARA SA [33, 34]), and this study will extend the evidence base to Zambia and build on prior research in several ways. First, we will monitor the effects over 24-months, providing new information about the durability of the intervention’s impact. Second, we are training peer leaders to deliver ZAIMARA which will increase relevance to participants [81], reduce the burden on the healthcare workforce through task shifting, and increase the potential for scalability and sustainability. Third, we will include a mental health component for peer leaders to address the often-overlooked challenge of job retention and satisfaction by attending to the mental well-being of those delivering the intervention. Finally, we will collect implementation and cost data in five sites, each distinct in population size and geography, engage key partners, capture facilitators and barriers to scale-up and sustainability, and inform health policy.

This study has additional strengths. We will collect both quantitative and qualitative data to allow for a deeper examination of participant and partner experiences and to strengthen ZAIMARA adoption, acceptability, appropriateness, and feasibility. The study draws on the unique expertise and partnership between U.S. and Zambian investigators ensuring that the intervention and the study design reflect genuine contributions by both sides. Indeed, prior to submitting any funding application, the intervention and study design were extensively vetted with key Ministry of Health staff, the MOH Adolescent Technical Working Group, and the CIDRZ adult and adolescent community advisory boards for concurrence. By engaging MF and AGYW together, ZAIMARA amplifies the impact of their relationship on SRH, because they can support one another in efforts to adopt prevention behavior. Lastly, ZAIMARA offers a combined behavioral and biomedical intervention package on-site, eliminating typical barriers to accessing HIV testing, PrEP, and STI testing and treatment.

Limitations of the study exist. We will rely on self-reports of MF-AGYW communication and relationships, and self-report data may suffer from recall and social desirability biases. To combat this risk, we will use a standardized self-administered CASI assessment system at all time points, with research assistants blinded to arm, and not peer leaders, helping AGYW and MF complete the survey. We will employ resident NHCs to recruit participants, and they may deliberately choose specific AGYW and MF in favor of their personal networks or possibly target AGYW who they deem are at ‘high risk’, hereby biasing our sample. To address the potential for selection bias, we will pair a research staff with NHC recruiters to ensure compliance with rigorous recruitment procedures. To minimize bias in assignment to condition, we will randomize MF-AGYW dyads and peer leaders to arm and evaluate any baseline differences that might influence effectiveness outcomes. Scalability and sustainability will be measured prospectively. Changes in leadership, country priorities and funding may affect the subsequent rollout and sustainability of the program. Finally, this trial will occur across five distinct geographical areas, and findings may not generalize to other districts/provinces.

In conclusion, this study will add to the evidence base testing family-based interventions for HIV prevention among AGYW and their scalability in low-resource settings. We expect the dual focus on AGYW and peer leaders’ health and retention, along with the incorporation of rigorous implementation science principles, to enhance ZAIMARA’s overall effectiveness, acceptability, feasibility, and sustainability. The proposed economic evaluation will guide resource allocation and promote sustainable HIV prevention strategies to end the epidemic in Zambia.

## Data Availability

A de-identified dataset will be made publicly available through the National Institute of Child Health and Human Development (NICHD) Data and Specimen Hub (DASH) https://dash.nichd.nih.gov.

## Abbreviations

AGYW: Adolescent Girls and Young Women
ART: Antiretroviral Therapy
CASI: Computerized Assisted Self-Interview
CDC: Centers for Disease Control and Prevention
CE: Cost Effectiveness
CIDRZ: Centre for Infectious Disease Research in Zambia
EPIS: Exploration, Preparation, Implementation, Sustainment
FGD: Focus Group Discussion
HIV: Human Immunodeficiency Virus
IDI: In-Depth Interview
IMARA: Informed, Motivated, Aware and Responsible Adolescents and Adults
IRLM: Implementation Research Logic Model
MF: Mother Figure
MoH: Ministry of Health
NHC: Neighborhood Health Committee
NIH: National Institutes of Health
PrEP: Pre-Exposure Prophylaxis
SRH: Sexual Reproductive Health
STI: Sexually Transmitted Infection
UNAIDS: Joint United Nations Programme on HIV/AIDS
ZAIMARA: Zambian Informed, Motivated, Aware and Responsible Adolescents and Adults

## References

[1] Zambia Ministry of Health. Adolescent health strategy 2017 to 2021. Zambia: Lusaka; 2017.

[2] Zambia Population-Based HIV Impact Assessment. 2021. Ministry of Health (MoH) Report. December 2022.

[3] UNICEF. All in to end the adolescent AIDS epidemic: A progress report. December 2016. Available at https://www.unaids.org/sites/default/files/media_asset/ALLIN2016ProgressReport_en.pdf

[4] Heri AB, Cavallaro FL, Ahmed N, Musheke MM, Matsui M. Changes over time in HIV testing and counselling uptake and associated factors among youth in Zambia: a cross-sectional analysis of demographic and health surveys from 2007 to 2018. BMC Public Health. 2021;21(1):456. 33676482 10.1186/s12889-021-10472-x PMC7937241

[5] Zambia Ministry of Health. 2022–2026 National Health Strategic Plan. Available from: https://www.moh.gov.zm/wp-content/uploads/2023/02/National-Health-Stratergic-Plan-for-Zambia-2022-to-2026-revised-February-2023-lower-resolution.pdf

[6] Moyo N, Müller JC. The influence of cultural practices on the HIV and AIDS pandemic in Zambia. HTS Teol Stud. 2011;67(3):412–7.

[7] Mhungu A, Sixsmith J, Burnett E. Adolescent girls and young women’s experiences of living with HIV in the context of patriarchal culture in sub-Saharan Africa: a scoping review. AIDS Behav. 2023;27(5):1365–79. 36318422 10.1007/s10461-022-03872-6 PMC10129999

[8] Sidamo NB, Kerbo AA, Gidebo KD, Wado YD. Socio-ecological analysis of barriers to access and utilization of adolescent sexual and reproductive health services in sub-Saharan Africa: a qualitative systematic review. Open Access J Contracept. 2023;14:103–18. 37398897 10.2147/OAJC.S411924 PMC10312343

[9] DiClemente RJ, Salazar LF, Crosby RA. A review of STD/HIV preventive interventions for adolescents: sustaining effects using an ecological approach. J Pediatr Psychol. 2007;32(8):888–906. 17726032 10.1093/jpepsy/jsm056

[10] Kuo C, Atujuna M, Mathews C, Stein DJ, Hoare J, Beardslee W, et al. Developing family interventions for adolescent HIV prevention in South Africa. AIDS Care. 2016;28(Suppl 1sup1):106–10. 26916841 10.1080/09540121.2016.1146396 PMC4828610

[11] Mbugua N. Factors inhibiting educated mothers in Kenya from giving meaningful sex-education to their daughters. Soc Sci Med. 2007;64(5):1079–89. 17258368 10.1016/j.socscimed.2006.10.008

[12] Peltzer K, Pengpid S. Sexuality of 16- to 17- year-old South Africans in the contexts of HIV/AIDS. Soc Behav Pers. 2006;34(3):239–56.

[13] Stevens JW. Smart and sassy: the strengths of Inner-City black girls. New York: Oxford University Press; 2002.

[14] Aronowitz T, Rennells RE, Todd E. Heterosocial behaviors in early adolescent African American girls: the role of mother-daughter relationships. J Fam Nurs. 2005;11(2):122–39. 16287822 10.1177/1074840705275466

[15] Donenberg GR, Emerson E, Mackesy-Amiti. Sexual risk among African American girls: psychopathology and mother-daughter relationships. J Consult Clin Psychol. 2011;79(2):153–8. 21319895 10.1037/a0022837 PMC3077919

[16] Emerson E, Donenberg GR, Wilson HW. Health-protective effects of attachment among African American girls in psychiatric care. J Fam Psychol. 2012;26(1):124–32. 22182334 10.1037/a0026352 PMC3716380

[17] Jaccard J, Dittus PJ, Gordon VV. Parent-adolescent congruency in reports of adolescents sexual behavior and in communications about sexual behavior. Child Dev. 1998;69(1):247–61. 9499570

[18] O’Donnell L, Stueve A, Durnan R, Myint-U A, Agronick G, San Doval A, et al. Parenting practices, parents’ underestimation of daughters’ risks, and alcohol and sexual behaviors of urban girls. J Adolesc Health. 2008;42:496–502. 18407045 10.1016/j.jadohealth.2007.10.008 PMC2376042

[19] Adu-Mireku S. Family communication about HIV/AIDS and sexual behavior among senior secondary school students in Accra, Ghana. Afr Health Sci. 2003;3:7–14. 12789082 PMC2141593

[20] Crichton J, Ibisomi L, Gyimah SO. Mother-daughter communication about sexual maturation, abstinence and unintended pregnancy: experiences from an informal settlement in Nairobi, Kenya. J Adolesc. 2012;35(1):21–30. 21783241 10.1016/j.adolescence.2011.06.008

[21] Dimbuene ZT, Defo BK. Risky sexual behaviour among unmarried young people in cameroon: another look at family environment. J Biosoc Sci. 2011;43(2):129–53. 21134307 10.1017/S0021932010000635

[22] Juma M, Alaii J, Askew A, Bartholomew L, Borne B. Community perspectives on parental/caregiver communication on reproductive health and HIV with adolescent orphans and Non-Orphans in Western Kenya. J Child Adolesc Behav. 2015;3:206.

[23] Puffer ES, Meade CS, Drabkin AS, Broverman SA, Ogwang-Odhiambo RA, Sikkema KJ. Individual- and family-level psychosocial correlates of HIV risk behavior among youth in rural Kenya. AIDS Behav. 2011;15(6):1264–74. 20945157 10.1007/s10461-010-9823-8 PMC3545696

[24] Soon CN, Kaida A, Nkala B, Dietrich J, Cescon A, Gray G, et al. Adolescent experiences of HIV and sexual health communication with parents and caregivers in Soweto, South Africa. SAHARA-J: Journal of Social Aspects of HIV/AIDS. 2013;10(3–4):163–9. 10.1080/17290376.2014.902769 24809230

[25] Isaksen KJ, Musonda P, Sandoy IF. Parent-child communication about sexual issues in Zambia: a cross sectional study of adolescent girls and their parents. BMC Public Health. 2020;20(1):1120. 32677930 10.1186/s12889-020-09218-y PMC7364553

[26] Butts SA, Kayukwa A, Langlie J, Rodriguez VJ, Alcaide ML, Chitalu N, et al. HIV knowledge and risk among Zambian adolescent and younger adolescent girls: challenges and solutions. Sex Educ. 2018;18(1):1–13. 31275062 10.1080/14681811.2017.1370368 PMC6606053

[27] Harrison A, Newell ML, Imrie J, Hoddinott G. HIV prevention for South African youth: which interventions work? A systematic review of current evidence. BMC Public Health. 2010;10:102. 20187957 10.1186/1471-2458-10-102 PMC2843660

[28] Denison JA, McCauley AP, Dunnett-Dagg WA, Lungu N, Sweat MD. The HIV testing experiences of adolescents in Ndola, Zambia: do families and friends matter? AIDS Care. 2008;20(1):101–5. 18278620 10.1080/09540120701427498

[29] Denison JA, McCauley AP, Lungu N, Sweat MD. Families matter: social relationships and adolescent HIV testing behaviors in Ndola, Zambia. Vulnerable Child Youth Stud. 2013;9(2):132–8.

[30] Roberts ST, Edwards P, Mulenga D, Chelwa N, Nyblade L, Brander C, et al. Family support for adolescent girls and young women living with HIV in Zambia: benefits, challenges, and recommendations for intervention development. J Assoc Nurses AIDS Care. 2021;32(2):160–73. 33332869 10.1097/JNC.0000000000000225 PMC7914154

[31] Nesamoney SN, Mejia-Guevara I, Cislaghi B, Weber AM, Mbizvo MT, Darmstadt GL. Social normative origins of the taboo gap and implications for adolescent risk for HIV infection in Zambia. Soc Sci Med. 2022;312: 115391. 36183540 10.1016/j.socscimed.2022.115391 PMC9582197

[32] Donenberg GR, Kendall AD, Emerson E, Fletcher FE, Bray BC, McCabe K. IMARA: a mother-daughter group randomized controlled trial to reduce sexually transmitted infections in Black/African-American adolescents. PLoS One. 2020;15(11): e0239650. 33137103 10.1371/journal.pone.0239650 PMC7605636

[33] Donenberg G, Merrill KG, Atujuna M, Emerson E, Bray B, Bekker LG. Mental health outcomes of a pilot 2-arm randomized controlled trial of a HIV-prevention program for South African adolescent girls and young women and their female caregivers. BMC Public Health. 2021;21(1):2189. 34847908 10.1186/s12889-021-12010-1 PMC8630514

[34] Merrill KG, Atujuna M, Emerson E, Blachman-Demner D, Bray BC, Bekker LG, et al. Preliminary effectiveness and implementation outcomes of the IMARA-South Africa sexual health intervention on adolescent girls and young women: a pilot randomized trial. PLOS Glob Public Health. 2023;3(2):e0001092. 36962830 10.1371/journal.pgph.0001092 PMC10022073

[35] Sissoko G, Kanguya T, Mwenge M, Sharma A, Chhun N, Yu SH et al. Using the framework for reporting adaptation and modifications-expanded (FRAME) to adapt an evidence-based HIV-prevention intervention for Zambian adolescent girls and young women. (Under Review). 2025. 10.1007/s10461-026-05033-5 PMC13400679 41591688

[36] Becan JE, Bartkowski JP, Knight DK, Wiley TRA, DiClemente R, Ducharme L, et al. A model for rigorously applying the exploration, preparation, implementation, sustainment (EPIS) framework in the design and measurement of a large scale collaborative multi-site study. Health Justice. 2018;6(1):9. 29654518 10.1186/s40352-018-0068-3 PMC5899075

[37] Moullin JC, Dickson KS, Stadnick NA, Rabin B, Aarons GA. Systematic review of the exploration, preparation, implementation, sustainment (EPIS) framework. Implement Sci. 2019;14(1): 1. 30611302 10.1186/s13012-018-0842-6 PMC6321673

[38] Carcone AI, Coyle K, Butame S, Harper GW, Aarons GA, Naar S. Using the exploration-preparation-implementation-sustainment (EPIS) framework to prepare for the implementation of evidence-based practices into adolescent HIV settings. AIDS Behav. 2022;26(12):4093–106. 36066763 10.1007/s10461-022-03735-0 PMC9643628

[39] Aarons GA, Hurlburt M, Horwitz SM. Advancing a conceptual model of evidence-based practice implementation in public service sectors. Adm Policy Ment Health. 2011;38(1):4–23. 21197565 10.1007/s10488-010-0327-7 PMC3025110

[40] Owuor RA, Mutungi K, Anyango R, Mwita CC. Prevalence of burnout among nurses in sub-Saharan Africa: a systematic review. JBI Evid Synth. 2020;18(6):1189–207. 32813372 10.11124/JBISRIR-D-19-00170

[41] Matovu JKB, Nambuusi A, Wanyenze RK, Serwadda D. Peer-leaders’ experiences and challenges in distributing HIV self-test kits in a rural fishing community, Rakai, Uganda. BMC Public Health. 2021;21(1):708. 33845811 10.1186/s12889-021-10804-x PMC8042983

[42] Rhee H, Love T, Harrington D, Walters L, Mammen J, Sloand E. Long-term effects of a peer-led asthma self-management program on asthma outcomes in adolescent peer leaders. Patient Educ Couns. 2021;104(6):1415–22. 33339656 10.1016/j.pec.2020.11.039 PMC8164959

[43] Jaguga F, Kwobah EK, Giusto A, Apondi E, Barasa J, Korir M, et al. Feasibility and acceptability of a peer provider delivered substance use screening and brief intervention program for youth in Kenya. BMC Public Health. 2023;23(1):2254. 37974158 10.1186/s12889-023-17146-w PMC10652467

[44] DiClemente RJ, Wingood GM. A randomized controlled trial of an HIV sexual risk-reduction intervention for young African-American women. JAMA. 1995;274(16):1271–6. 7563531

[45] DiClemente RJ, Wingood GM, Harrington KF, Lang DL, Davies SL, Hook EW 3, et al. Efficacy of an HIV prevention intervention for African American adolescent girls: a randomized controlled trial. JAMA. 2004;292(2):171–9. 15249566 10.1001/jama.292.2.171

[46] Brown LK, Hadley W, Donenberg GR, DiClemente RJ, Lescano C, Lang DM, et al. Project STYLE: a multisite RCT for HIV prevention among youths in mental health treatment. Psychiatr Serv. 2014;65(3):338–44. 24382603 10.1176/appi.ps.201300095 PMC9215702

[47] Donenberg GR, Brown LK, Hadley W, Kapungu C, Lescano C. Family-based HIV-prevention program for adolescents with psychiatric disorders. In: Pequegnat W, Bell CC, editors. Families and HIV/AIDS: culture and contextual issues in prevention and treatment (pp. 261–278). New York: Springer; 2012.

[48] Donenberg G, Emerson E, Bryant F, Wilson H, Weber-Shifrin E. Understanding AIDS-risk behavior among adolescents in psychiatric care: links to psychopathology and peer relationships. J Am Acad Child Adolesc Psychiatry. 2001;40(6):642–53. 11392341 10.1097/00004583-200106000-00008 PMC1201503

[49] Gorsky RD. A method to measure the costs of counseling for HIV prevention. Public Health Rep. 1996;111(supplement 1):115–22. 8862166 PMC1382052

[50] Gorsky RD, Teutsch SM. Assessing the effectiveness of disease and injury prevention programs: costs and consequences. MMWR. 1995;RR–10:1–10. 7565538

[51] Kalichman SC, Simbayi L. Traditional beliefs about the cause of AIDS and AIDS-related stigma in South Africa. AIDS Care. 2004;16(5):572–80. 15223526 10.1080/09540120410001716360

[52] Hoff CC, Chakravarty D, Bircher AE, Campbell CK, Grisham K, Neilands TB, et al. Attitudes towards PrEP and anticipated condom use among concordant HIV-negative and HIV-discordant male couples. AIDS Patient Care STDS. 2015;29(7):408–17. 26057304 10.1089/apc.2014.0315 PMC4504342

[53] Margolies PJ, Weintraub S. The revised 56-item CRPBI as a research instrument: reliability and factor structure. J Clin Psychol. 1977;33:473–6.

[54] Dutra R, Miller KS, Forehand R. The process and content of sexual communication with adolescents in two-parent families: associations with sexual risk-taking behavior. AIDS Behav. 1999;3(1):59–66.

[55] Miller KS, Levin ML, Whitaker DJ, Xu X. Patterns of condom use among adolescents: the impact of mother-adolescent communication. Am J Public Health. 1998;88(10):1542–4. 9772860 10.2105/ajph.88.10.1542 PMC1508458

[56] Miller KS, Kotchick BA, Dorsey S, Forehand R, Ham AY. Family communication about sex: what are parents saying and are their adolescents listening? Fam Plann Perspect. 1998;30(5):218–22. 9782044

[57] Ali GC, Ryan G, De Silva MJ. Validated screening tools for common mental disorders in low and middle income countries: a systematic review. PLoS One. 2016;11(6): e0156939. 27310297 10.1371/journal.pone.0156939 PMC4911088

[58] Kroenke K, Spitzer RL, Williams JB. The PHQ-9: validity of a brief depression severity measure. J Gen Intern Med. 2001;16(9):606–13. 11556941 10.1046/j.1525-1497.2001.016009606.x PMC1495268

[59] Mughal AY, Devadas J, Ardman E, Levis B, Go VF, Gaynes BN. A systematic review of validated screening tools for anxiety disorders and PTSD in low to middle income countries. BMC Psychiatry. 2020;20(1):338. 32605551 10.1186/s12888-020-02753-3 PMC7325104

[60] Poudyal A, Lovero K, Merrill K, Wilson EC, Grinsztejn B, Jalil EM et al. Psychometric evaluation of measures for rapid identification of mental health problems among adolescents and young adults affected by HIV across Resource-Constrained settings in Africa and Brazil. Under Review (2025).

[61] Inoue S, Chitambi C, Vinikoor MJ, Kanguya T, Murray LK, Sharma A, et al. Testing the validity of the AUDIT-C and AUDIT-3 to detect unhealthy alcohol use among high-risk populations in Zambia: a secondary analysis from two randomized trials. Drug Alcohol Depend. 2021;229(Pt A): 109156. 34773884 10.1016/j.drugalcdep.2021.109156 PMC8671251

[62] Kane JC, Murray LK, Bass JK, Johnson RM, Bolton P. Validation of a substance and alcohol use assessment instrument among orphans and vulnerable children in Zambia using audio computer assisted self-interviewing (ACASI). Drug Alcohol Depend. 2016;166:85–92. 27402551 10.1016/j.drugalcdep.2016.06.026 PMC4983530

[63] Levola J, Aalto M. Screening for at-risk drinking in a population reporting symptoms of depression: a validation of the AUDIT, AUDIT-C, and AUDIT-3. Alcohol Clin Exp Res. 2015;39(7):1186–92. 26058472 10.1111/acer.12763

[64] What Works To Prevent Violence. A global programme to prevent violence against women and girls. Standard Outcomes for Assessment of Intimate Partner Violence; 2015.

[65] el-Bassel N, Ivanoff A, Schilling RF, Gilbert L, Borne D, Chen DR. Preventing HIV/AIDS in drug-abusing incarcerated women through skills building and social support enhancement: preliminary outcomes. Soc Work Res. 1995;19(3):131–41. 10172402

[66] Brinckley MM, Calabria B, Walker J, Thurber KA, Lovett R. Reliability, validity, and clinical utility of a culturally modified Kessler scale (MK-K5) in the aboriginal and Torres Strait Islander population. BMC Public Health. 2021;21(1):1111. 34112127 10.1186/s12889-021-11138-4 PMC8194217

[67] Batista P, Neves-Amado J, Pereira A, Amado J. Fantastic lifestyle questionnaire from 1983 until 2022: a review. Health Promot Perspect. 2023;13(2):88–98. 37600548 10.34172/hpp.2023.11 PMC10439457

[68] Alpern R, Canavan ME, Thompson JT, McNatt Z, Tatek D, Lindfield T, et al. Development of a brief instrument for assessing healthcare employee satisfaction in a low-income setting. PLoS One. 2013;8(11): e79053. 24223878 10.1371/journal.pone.0079053 PMC3818514

[69] Tietjen-Smith TM, Kimbrough S, Balkin RS. Development and validation of the Sex Education Confidence Scale (SECS). Retrieved from http://www.scientificjournals.org/journals2008/articles/1406.pdf Journal of Education and Human Development. 2008;2(2).

[70] Chang E, Cohen J, Koethe B, Smith K, Bir A. Measuring job satisfaction among healthcare staff in the United States: a confirmatory factor analysis of the satisfaction of employees in health care (SEHC) survey. Int J Qual Health Care. 2017;29(2):262–8. 28339641 10.1093/intqhc/mzx012

[71] Spitzer RL, Kroenke K, Williams JB, Lowe B. A brief measure for assessing generalized anxiety disorder: the GAD-7. Arch Intern Med. 2006;166(10):1092–7. 16717171 10.1001/archinte.166.10.1092

[72] Prins A, Ouimette P, Kimerling R, Cameron RP, Hugelshofer DS, Shaw-Hegwer J, et al. The primary care PTSD screen (PC-PTSD): development and operating characteristics. Prim Care Psychiatry. 2003;9(1):9–14.

[73] Proctor E, Silmere H, Raghavan R, Hovmand P, Aarons G, Bunger A, et al. Outcomes for implementation research: conceptual distinctions, measurement challenges, and research agenda. Adm Policy Ment Health. 2011;38(2):65–76. 20957426 10.1007/s10488-010-0319-7 PMC3068522

[74] Donenberg GR, Emerson E, Bryant FB, Wilson H, Weber-Shifrin E. Understanding AIDS-risk behavior among adolescents in psychiatric care: links to psychopathology and peer relationships. J Am Acad Child Adolesc Psychiatry. 2001;40(6):642–53. 11392341 10.1097/00004583-200106000-00008 PMC1201503

[75] Glasgow RE, Harden SM, Gaglio B, Rabin B, Smith ML, Porter GC, et al. Re-aim planning and evaluation framework: adapting to new science and practice with a 20-year review. Front Public Health. 2019;7:64. 30984733 10.3389/fpubh.2019.00064 PMC6450067

[76] Harris PA, Taylor R, Minor BL, Elliott V, Fernandez M, O’Neal L, et al. The REDCap consortium: Building an international community of software platform partners. J Biomed Inf. 2019;95:103208. 10.1016/j.jbi.2019.103208 PMC7254481 31078660

[77] Harris PA, Taylor R, Thielke R, Payne J, Gonzalez N, Conde JG. Research electronic data capture (REDCap)--a metadata-driven methodology and workflow process for providing translational research informatics support. J Biomed Inform. 2009;42(2):377–81. 18929686 10.1016/j.jbi.2008.08.010 PMC2700030

[78] Baron RM, Kenny DA. The moderator-mediator variable distinction in social psychological research: conceptual, strategic, and statistical considerations. J Pers Soc Psychol. 1986;51(6):1173–82. 3806354 10.1037//0022-3514.51.6.1173

[79] Cohen J, Cohen P. Applied multiple regression/correlation analysis for the behavioral sciences. Hillsdale, NJ: Erlbaum; 1983.

[80] Joint United Nations Programme on HIV/AIDS (UNAIDS). 2024 Global AIDS update. Thematic briefing note: HIV and adolescent girls and young women. Accessible at https://www.unaids.org/sites/default/files/media_asset/2024-unaids-global-aids-update-adolescent-girls-young-women_en.pdf

[81] Wiebel WW, Rockville. Md: National Institute on Drug Abuse, Division of Applied Research, Community Research Branch.

